# Synthesis of ecologically effective adsorbent from theba pisana snails for enhanced adsorption of Pb and Fe

**DOI:** 10.1038/s41598-025-22770-0

**Published:** 2025-11-11

**Authors:** Amany R. Salem, Ibrahim Hegazy, Walaa A. Kassab, Hesham A. M. Ibrahim

**Affiliations:** 1 Nuclear Materials Authority, El -Maadi, P.O. Box 530, Cairo, Egypt; 2 The Holding Company for Drinking Water in Greater Cairo (10Th of Ramadan Authority), Cairo, Egypt; 3 Department of Agricultural Zoology and Nematology, Faculty of Agriculture, Al-Azhar University, Assiut Branch, Assiut, 71524 Egypt

**Keywords:** Land snail pests; calcined snails, Metal ions Adsorption; isotherms, Kinetics, Error function, Thermodynamics, Environmental sciences, Environmental social sciences

## Abstract

This study introduces an innovative approach to repurposing agricultural pest snails as effective adsorbents for heavy metal removal, offering a sustainable and eco-friendly solution for wastewater treatment. The recovery of metal ions such as lead and ferrous ions (Pb (II), Fe (II)) from contaminated and aqueous solutions has been investigated using the land snail, Theba pisana, by a single thermal decomposition method. The produced material was successful in removing heavy metals from solutions, as evidenced by the improved adsorption effectiveness of the generated adsorbent. By adjusting several physicochemical factors, including time, pH, dosage, beginning concentration, and temperature, batch experiment research has been clarified. The optimization of lead and iron ion adsorption onto prepared adsorbent was performed considering the factors of fabricated adsorbent pH (1–6); concentrations (50–400 mg/L); adsorbent dose (0.005–0.05 g), time (5–120 min), and temperature (298–318 K).The optimum time and temperature were found to be 45 min and room temperature. FTIR, SEM with EDX, and other Spectro analytical methods were used to analyze the as-synthesized adsorbent. To ascertain which of the Freundlich and Langmuir isotherm models most accurately captured the reported adsorption equilibrium data, these models were employed. To further clarify the adsorption mechanism, the rate of adsorption was also investigated using a range of linear kinetic models (pseudo first order, pseudo second order and intraparticle diffusion), which showed pseudo-second-order behavior and indicated that two processes are essential to the adsorption process. In order to comprehend the nature of the sorption process, the mechanism of the adsorption process was shown by computing the values of ΔG^o^, ΔH^o^, and ΔS^o^ using thermodynamic parameters. Consequently, adsorbent was a readily accessible, affordable, and ecologically suitable adsorbent for the removal of metal ions. An option for the adsorption of metal ions from polluted wastewater is the produced adsorbent.

**Supplementary Information:**

The online version contains supplementary material available at 10.1038/s41598-025-22770-0.

## Introduction

Heavy metal (HM) poisoning of the environment has drawn a lot of attention because of the harmful effects that HMs have on humans, animals, and plants. Therefore, from an environmental and biological perspective, it is imperative that these harmful substances (HMs) be cleaned up from the environment^1^. Because they are not biodegradable or thermodegradable, heavy metals easily build up in living things and have detrimental effects on ecosystems and human health. Even in tiny concentrations, they are extremely difficult to naturally remove from the environment due to their persistence. Population expansion and the ensuing increase in heavy metal contamination of the environment, which has a significant impact on human health, are linked to intensive anthropogenic activities including residential, transportation, and industrial development^2^. Numerous nanoparticles (NPs), including metals and their oxides, carbon compounds, zeolites, and bimetallic NPs, have been reported to be effective in cleaning up environments contaminated by heavy metals^3^.

One of the most hazardous heavy metals is lead. It is extensively utilized in several significant industrial processes, including the production of explosives, pigments, fuels, printing, storage batteries, and photographic materials. Lead is known to be hazardous to humans; it may build up in the skeletal system because it substitutes calcium^4^. There are major threats to the environment and human health from this heavy metal, which is also regarded as one of the most hazardous. Due to its numerous industrial uses, this element, which is naturally present in the Earth’s crust, has been utilized throughout history. Numerous studies have examined the negative consequences of lead exposure, and it is commonly known that even low amounts of lead may result in major health issues^5^. A quantity of more than 0.07 mg/g of lead in drinking water can have several negative health consequences on the kidneys, brain, and neurological system. Lead has the ability to pollute soil, which might have long-term consequences and endanger human health as well as ecosystems^6^.

The neurological system, which can cause memory and concentration issues; adverse effects on children’s behavior and cognitive development; elevated risk of cardiovascular diseases, such as high blood pressure and coronary artery disease; and effects on the gastrointestinal system are just a few of the many ways that lead affects human health, which can include respiratory irritation, which can result in symptoms like coughing, breathing difficulties, and mucous membrane irritation; symptoms including nausea, constipation, and stomach discomfort; and impacts on reproduction, which can affect both men’s and women’s reproductive systems. Negative effects on sexual development and fertility may arise from this^7^. Additionally, lead has detrimental effects on the agronomy and productivity of plants. For example, it can interfere with essential biochemical processes by disrupting enzymatic activity^8,9^.

One of these heavy metals is iron, which is the second most common metal element in the crust of the Earth and is mostly found in natural water in the two oxidation forms of Fe (II) and Fe (III). While its oxidized forms, methemoglobin and metmyoglobin, which contain Fe(III), will not bind oxygen, hemoglobin and myoglobin require Fe(II) for the appropriate delivery and storage of oxygen^10^. Environmental redox equilibria depend on the redox pair Fe (II) ∕Fe (III). When combined with Fe (III) hydroxides and oxides, dissolved iron in natural fluids can take the form of the Fe (II) ion^11^. Thus, a comprehensive experimental and theoretical investigation was conducted on the reactions between dissolved Fe (II) ions and Fe (III) oxides^11–13^. It is exceedingly difficult to experimentally investigate Fe (II) adsorption on metal (hydro)oxides under well-defined circumstances. Because the pH range where the adsorption takes place may have a very high rate of ferrous ion oxidation by dissolved oxygen^11^. Among the most effective and eco-friendly activators, Fe (II) is frequently used in Fenton reactions^14,15^. Fe is an important component of several enzymes and hemoglobin^16^. Deposition of insoluble iron in soil voids can block soil pores, decreasing the soil’s long-term permeability. Accordingly, sections of land that have high iron levels in their soils may become unproductive^17^. In the sediments of various water surface levels, iron easily complexes with sulphates. When iron is present in drinking water, the main issue is that it tastes bad. Even at low quantities of around 1.8 mg/l, the taste of iron in drinking water is plainly detectable^18^.

Egg shells, oyster shells, gastropod shells, crab shells, scallop shells, and mussel shells are examples of carbonate shell biowaste that can be used for Pd and iron adsorption^19^. Because of their high calcium carbonate (CaCO_3_) content, the shells may be calcined to create CaO molecules, which are useful for absorbing toxic substances like metals^20^. Water filtration, biodiesel manufacturing, and petroleum refining are just a few of the sectors that depend on calcium oxide (CaO), sometimes referred to as quicklime or burned lime. CaO is a crucial component in many applications and is usually made by heating CaCO_3_ materials, like limestone or seashells, at 825 °C^21^. The majority of heavy metals may be immobilized using calcium-based adsorbents (such CaO and CaCO_3_) due to their affordable benefits and easily accessible raw ingredients^22^.

Since calcium oxide (CaO) nanocomponents are safe for human use and have important structural and distinctive optical qualities, they are suggested to help in a number of fields, including medicine delivery, environmental cleanup, electronic utilities, sensors, and catalysis^23^. CaO is a metal oxide that is found in alkaline earth compounds. It is thought to be useful as an effective chemo-sorbent for HCl, toxic gasses, halogenated chemicals, and even compounds that include phosphorus. Typically, calcium and barium oxides are used for these functions. Calcium carbonate waste shells are thermally deteriorated to create CaO, an alkaline white, crystalline powder. Chemically produced CaO is used in a variety of industrial utilities for catalytic purposes as an adsorbent, hazardous cleanup agent, etc. Additionally, waste shell is a significant source of calcium oxide (CaO), which makes about 95% of calcium carbonate. As a result, discarded shells add significantly to the amount of waste material that is dumped in surrounding landfills without any kind of pre-treatment, which further affects the soil and causes unpleasant odors. Therefore, CaO nanoparticles produced from such shells have been effectively used in fuel cell-based applications, catalysts, biomaterials, biodiesel production, hazardous pollutant sequestration, and dielectrics. Because of their capacity to influence material contacts, Ca^2+^ is regarded as the most prevalent ionic form of the calcium ion in the arrangement that is crucial to material adhesion. Ca^2+^ was applied directly to the naphthalene surface during the adsorption cycle^24^. By computing the associated adsorption energy, the calcium ion adsorption instrument on the naphthalene surface was investigated. These ionic species could modify the surface physicochemical characteristics of the particles, basically. In the cation adsorption system, numerous studies have highlighted that a substantial portion of pH influences cation adsorption on hydrous metal oxides^25^.

Consequently, a number of techniques have been developed to remove heavy metal ions, including as adsorption, membrane technologies, electrochemical technologies, ion exchange, precipitation, coagulation and flocculation, and others^26^. Because of a number of technological and economic considerations, only a small number of treatment techniques are employed. Although these methods are capable of eliminating a variety of contaminants from water and wastewater, they have some disadvantages (expensive, complicated, inefficient, etc.). Interestingly, the most efficient technique for eliminating heavy metals was found to be the adsorption approach, which is thought to be the safest, cleanest, most practicable, and technically possible procedure^27^. Adsorption has been acknowledged as a low-energy, easy, and cost-effective method for recovering metal contaminants from aqueous solutions^28^.When it comes to environmental issues, the use of nanoparticles is quite desired^29^, metal protection from corrosion^30^, optoelectronics^31^, energy and storage devices^32^, biological sectors^33^, and analytical sciences^34^. Because of their ease of synthesis and functionalization, large active surface area, chemical variety, mechanical and thermal durability, and physical tunability, nanomaterials have been utilized more and more recently to purify water. They are classified as photocatalytic materials, adsorbents, membranes, etc. in contrast to conventional materials^35,36^. Researchers from all around the world may be able to investigate the possibilities of using readily available and safe sources, including bacteria, actinomycetes, yeast, fungus, algae, herbs, and plants, to synthesize NPs using green chemistry and bioprocesses^37^.

Regarding the target organisms to be used in this study, gastropoda, the largest class within the phylum Mollusca, inhabits marine, freshwater, and terrestrial ecosystems. Among terrestrial gastropods, the terrestrial snails are recognized as significant agricultural pests inflicting significant harm to a range of crops, such as ornamental plants, fruits, and vegetables, by feeding on leaves, fruits, tubers, buds, and roots^38,39^ . In Egypt, these snails are particularly prevalent in the Delta region and the northern Mediterranean coast^40,41^ and have also spread into Upper Egypt^39,42^. Economic losses are attributed not only to direct feeding but also to contamination from snail bodies, feces, and slime, which can degrade product quality and thus a decrease in its marketing value One of these snails is the terrestrial snail Theba pisana, which has grown to be a significant agricultural pest that damages crops in many locations throughout Egypt and whose population has to be controlled^43^.

In many regions of the world, the helicid land snail T. pisana poses a significant threat to a variety of crops, including cereals, vegetables, grapevines, shrubs, and ornamental plants. Its reputation as a troublesome pest is a result of its gregarious behavior, quick rate of reproduction, and difficulties under traditional management. However, many species of molluscs, as invertebrates, play a role as bioindicators for monitoring environmental pollution in ecosystems, as they can be affected by and accumulate by many metals, whether they are terrestrial or freshwater molluscs. Among terrestrial invertebrates, there are various herbivorous land snail species, including the helicid white garden snail T. pisana. that are employed as sensitive markers to identify climate shifts and chemical contamination. Helix snails are good for tracking trace metals, agrochemicals, urban pollutants, and electromagnetic radiation because they may collect several chemical classes^44^. Biomarkers offer valuable early indicators of environmental stress in biological indicator species, providing insight into ecosystem threats in polluted areas^45,46^. Interactions between heavy metals and antioxidant defense systems are critical in understanding an organism’s ecotoxicological responses to its environment^47^. The sustainability and affordability of the new, environmentally friendly process for creating CaO NPs from T. pisana shell are highlighted in this work. In addition to producing valuable nanomaterials, this method recycles T. pisana shell waste with no harm to the environment. By eliminating hazardous chemicals and intricate processes, the synthesis approach is both economical and environmentally friendly. It also works at a relatively low temperature that is adequate to produce the required CaO NPs characteristics. This low-temperature process is economically feasible for large-scale manufacturing since it uses less energy than conventional techniques, requires less equipment, and has shorter processing periods.

Overall, this study provides a workable approach to using the pests’ adsorbent to efficiently extract MI from wastewater. This study aimed to critically assess the usefulness of the terrestrial snail T. pisana as a sensitive biomarker for ecosystem pollution and to particularly identify the harmful effects of (Studied components) on this snail. In addition, this study sought to determine the binding processes and characterize the chemicals that result from adsorption at calcined snails.

## Material and method

### Chemicals, material and equipment

All chemicals and reagentsof analytical grade were used in this research work and purchased from Merck-(Germany) and Sigma-Aldrich-Chemical-Co. (USA). To prepare the polluted solution, lead nitrate (Pb (NO_3_)_2_) and ammonium ferrous sulfate ((NH_4_)_2_SO_4_·Fe (SO_4_) ·6H_2_O.) were utilized. This yields a 1000 mg L^−1^ stock solution.

### Collecting experimental snails

For use as test subjects, adult specimens of the herbivorous terrestrial snail Theba pisana (Muller) of comparable size and age were gathered. Ali and Robinson’s identification of these species^48^. They were collected from different orchards within the geographical range in Faisal region, Suez Governorate, Egypt. The samples were placed in clean plastic bags and transported to the Nuclear Materials Authority (NMA) laboratories. Photograph of the white garden snails, T. pisana, and the damage it causes to some crops as shown in Fig. S1(a).

### Synthesis of adsorbent

**Fig. 1:**
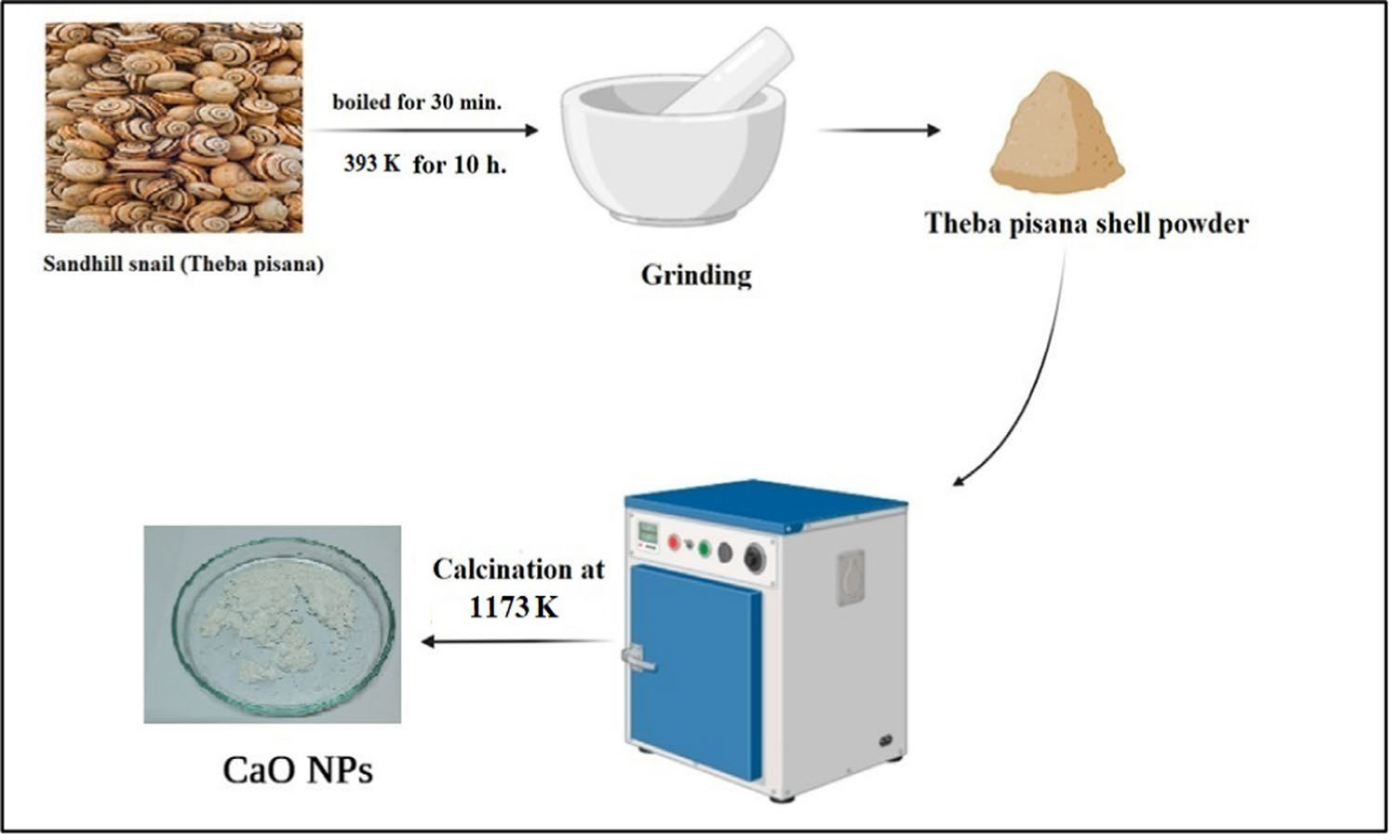
A schematic figure for conversion of snail to white snail calcium oxide.

Crops are seriously threatened by terrestrial snails because they severely harm the leaves, fruits, and roots. The extracted waste sandhill snail of T. pisana was thoroughly cleaned with double-distilled water and then cooked for 30 min in regular water to get rid of any remaining contaminants. Sandhill snails that had been well cleaned were then placed in a hot oven set at 393 K for ten hours. To make the dried snail shells powder, they were crushed using a milling machine. Additionally, it is calcined in a heating furnace at 1073 K. Calcium carbonate’s (CaCO_3_) full conversion occluded in Sandhill snails to CaO occurs when 1173 K. is the applied calcination temperature level. To get fine CaO nanoparticles, it is further cleaned and dried. Photographic of the white garden snail calcium oxide is shown in Fig. 1 and the schematic design illustrates how the calcium oxide was created by thermal breakdown was summarized in Fig. 1.

### Adsorbent characterization

The surface morphology and chemical composition were evaluated using a JEOL scanning electron microscope (SEM, JSM-5400LV, Japan) fitted with an energy dispersive X-ray spectroscopy (EDX). Fourier transform infrared spectroscopy (FT-IR) in the 4000–400 cm^−1^ range were utilized to examine the chemical functional groups of the prepared adsorbents before and after loading with different metal ions using a Vertex 70 FTIR-FT Raman spectrometer. BET surface area analysis was measured with the help of MICROTRAC, MRB, BELSORP MINIX(Japan), S/N:10,039 Weight 31kg, Power:AC100-240V,50/60Hz,10A, Microtrac BEL, Egyptian Desert Institute and The Nicomp N3000 DLS Particle Size Analyzer The Nicomp® dynamic light scattering (DLS) system measures the size of particles from 0.3 to 10 µm, in Egyptian Desalination Research Center of Excellence (EDRC) at the Desert Research Center Egyptian desert institute.

### Adsorption–desorption studies on snail* Theba pisana* powder

The ability of heavy metal ions to bind to the produced adsorbent was assessed in batch adsorption experiments. Metal ion sorption on the adsorbent was carried out using the batch sorption technique in 15 mL Oak Ridge tubes, which were then allowed to equilibrate for two hours at room temperature (298 K). For this experiment, a 1000 mg/L stock standard solution of lead and ferrous ions was prepared by dissolving specific weight lead and ferrous salts in ultrapure water. Lower lead and ferrous ions concentrations were achieved by diluting the ultrapure water-based synthetic stock solutions. The effects of different factors during this work on adsorption process, such as pH, adsorbent dose, initial concentration of lead and ferrous ions and temperature were investigated using batch technique. A 25 mL beaker containing 15 mL of lead and ferrous ions concentration-controlled solution was filled with a set amount of adsorbent (0.03 mg) for each adsorption test. The solution’s original pH was changed from 1 to 6 to get the correct result. The vessel was then agitated for a predetermined amount of time (t) while maintaining a steady temperature (T) at 200 rpm. The concentrations were measured following the use of filter paper to filter the filtrate solution for lead and ferrous ions. The measured remained lead and ferrous ions concentration was used to determine and evaluate the adsorption efficiency and capacity .The following three mathematical Eqs. (1, 2, 3) were used to evaluate the adsorption efficiency (% M) for lead and ferrous ions, the adsorption capacity of lead and ferrous ions at equilibrium (q_e_), and the at any time (qt)^49–52^:

1 $$\% M = \left( {1 - \frac{{C_{e} }}{{C_{0} }}} \right) \times 100$$

2 $$q_{t} = \frac{{\left( {C_{O} - C_{t} } \right)}}{m} \times V_{L}$$

3 $$q_{e} = \frac{{\left( {C_{O} - C_{e} } \right)}}{m} \times V_{L}$$

Here, the initial concentration and the equilibrium concentration of lead and ferrous ions is denoted by C_o_ (mg/L) and by C_e_ (mg/L), respectively , the amounts adsorbed at equilibrium and time, respectively, are denoted by q_e_ and q_t_, with the unit being (mg/g); the volume of lead and iron solution is denoted by V (L), and the mass of the prepared adsorbent is denoted by m (g). For this batch adsorption study, each experiment was carried out in triplicate.

### Investigations of adsorption thermodynamics

Gibbs free energy (ΔG°); entropy (ΔS°); and enthalpy (ΔH°) were among the thermodynamic characteristics that were evaluated in order to ascertain the type of HMI adsorption utilizing an adsorbent. To assess the thermodynamic parameters, the following formulas were utilized^53^:

4 $$\Delta {\varvec{G}}^\circ = - {\varvec{RT}}\;{\varvec{lnk}}_{{\varvec{C}}}$$

5 $${\varvec{k}}_{{\varvec{C}}} = \left( { {\varvec{q}}_{{\varvec{e}}} } \right)/\left( {{\varvec{C}}_{{\varvec{e}}} } \right)$$

6 $$\ln \;{\varvec{k}}_{{\varvec{C}}} = \frac{{\Delta {\varvec{S}}^\circ }}{{\varvec{R}}} - \frac{{\Delta {\varvec{H}}^\circ }}{{{\varvec{RT}}}}$$

Plotting of ln K_C_ versus 1/T is used to assess ΔH^o^ and ΔS^o^; k_C_ is a constant related to the equilibrium that may be determined using Eq. 6; T is the absolute temperature in Kelvin (K); The gas constant; R; has a value of 8.314 J / (mol. K). The uptake capacity of Pb (II) and Fe (II) ions adsorbed at equilibrium is q_e_ in mg/g, whereas the concentration of MI ions at equilibrium is C_e_ in mg/L.

### Statistical analysis

The Origin Program was used for all model fitting. The data displayed in bar plots is the mean of three replicates plus standard error, which is displayed as error bars.

## Result and discussion

### Characterization

#### Scanning electron microscopic (SEM) analysis

**Fig. 2:**
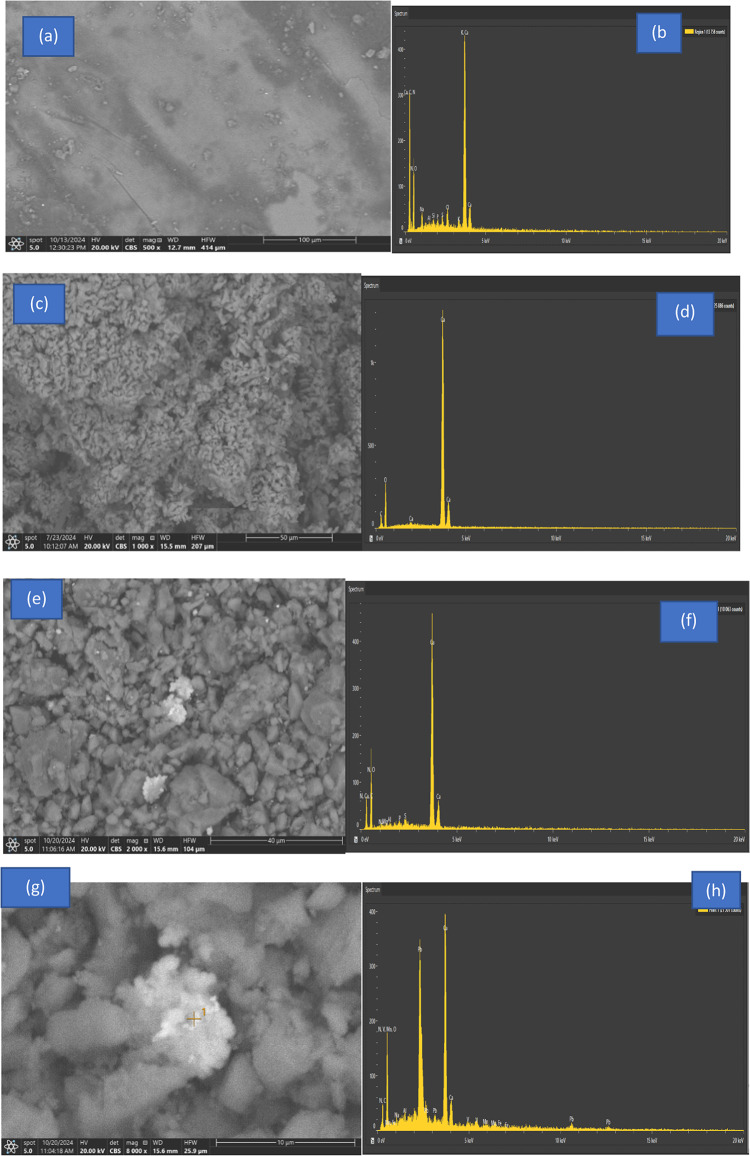
(**a**) SEM images and (**b**) EDX spectra of Theba pisana Snails., (**c**)SEM images and(**d**) EDX spectra of Theba pisana Snails after calcination process (**e**) SEM micrograph images and (**f**)EDX spectra of loaded fabricated adsorbent after adsorption and application to real waste solutions, Another spot (**g**)SEM micrograph images and (**h**)EDX spectra of loaded fabricated adsorbent after adsorption and application to real waste solutions.

The form, texture, morphologies, size, and arrangement of snail nanoparticles (NPs) on a diameter scale were all examined using SEM. It demonstrated that the NPs were massive collections of little particle materials that resembled foamy materials and sponges. Before and after the snails are heated, the CaO nanoparticles have different appearances. The CaO sorbent sample's SEM visual is displayed in Fig. 2(a). The bright areas of the surfaces in the image show high electron emission when exposed to the electron beam of the SEM. This illustrates the high surface area-to-volume ratio of the bright regions of surfaces. As can be observed in the micrograph, the manufactured particles were made up of spherically shaped grains that had aggregated together as shown in Fig. 2(c). These microscopic particles clumped together, revealing the polycrystalline structure of CaO NPs. However, after metal (II) ions were adsorbed, as indicated in Fig. 2(e) and (g), the outermost layer of CaO NPs changed and was deposited onto its surface.

**Table 1:** Thermodynamic parameters for the adsorption of lead and ferrous ions with the bio composite adsorbent.

Plot	Fe (II)	Pb (II)	Temp, K	ΔG^o^ (Fe (II))	ΔG^o^ (Pb (II))
ΔH^◦^	− 35.28588081	− 29.36226065	298	− 3.841716732	− 3.064168456
ΔS^◦^	− 0.10481676	− 0.087773558	303	− 3.652781534	− 2.835192393
R-Square	0.94719	0.96378	308	− 3.1669929	− 2.546941964
Adj. R-Square	0.92958	0.95171	313	− 2.654693711	− 1.842897229
Residual Sum of Squares	0.02796	0.01305	318	− 1.694949243	− 1.350547347

**Table 2:** Parameters of linear adsorption kinetic modes of calcined adsorbent on the recovery of lead and ferrous ions.

Kinetic Models	parameters	Pb (II)	Fe (II)
PFO	Residual Sum of Squares	1.86812	2.38295
	Pearson’s r	− 0.8546	− 0.85388
	R-Square	0.73034	0.72911
	Adj. R-Square	0.69182	0.69041
	k_1_	0.046336	0.052163
	q_e_	11.71764	20.49463
PSO	Residual Sum of Squares	4.45 × 10^−4^	0.00186
	Pearson’s r	0.99989	0.99945
	R-Square	0.99978	0.9989
	Adj. R-Square	0.99974	0.99874
	qe	79.36507937	86.05852
	k_2_	0.00597516	0.002979
RIDE	C	55.30601	50.52296
	k_p_	2.53405	3.55537
	Residual Sum of Squares	209.8501	172.924
	Pearson’s r	0.82986	0.91699

**Table 3:** Linear Freundlich and Langmuir isotherm for adsorption of for lead (II) and ferrous ions (II) on prepared calcined adsorbent.

Linear Models	Parameters	Pb (II)	Fe (II)
Langmuir	q_m_	1.16 × 10^+2^	1.38 × 10^+2^
	K_L_	6.51 × 10^−2^	4.42 × 10^−2^
	R-Square	0.99508	0.998
	Residual Sum of Squares	0.00897	1.85 × 10^−3^
	Pearson’s	0.99754	0.999
	Adj. R-Square	0.99385	0.9975
Freundlich	n	2.90E + 00	2.24E + 00
	K_f_	20.02717297	14.63843
	Pearson’s r	0.98834	0.9756
	R-Square	0.97681	0.9518
	Adj. R-Square	0.97102	0.93975
	Residual Sum of Squares	0.03739	0.09206

##### EDX

EDX analysis of Theba pisana Snail before and after calcination effect is shown in Table S1–S2. The elemental analysis of the adsorbent was investigated using EDX spectroscopy both before and during the adsorptive action of lead and iron as shown in Fig (b, d). Figure 2(d) displays the EDX analysis of ions adsorption on CaO with compositions of O, Ca, and C of 62.6%, 32.4%, and 5.1%, respectively. Applying the optimum condition on a real sample was also shown in Table S3–S4. As seen in Fig. 2(f, h) and Table S3–S4, the outermost layer of the CaO NPs altered after the metal ions were adsorbed. In accordance with the successful bonding of the adsorbed metal ions, it was found that metal ions are coated on the adsorbent surface. In Fig. 2(f, h), the EDX analysis of metal ion adsorption on CaO is displayed together with the adsorbed ions' composition of O, Ca, and C.

##### FTIR

**Fig. 3:**
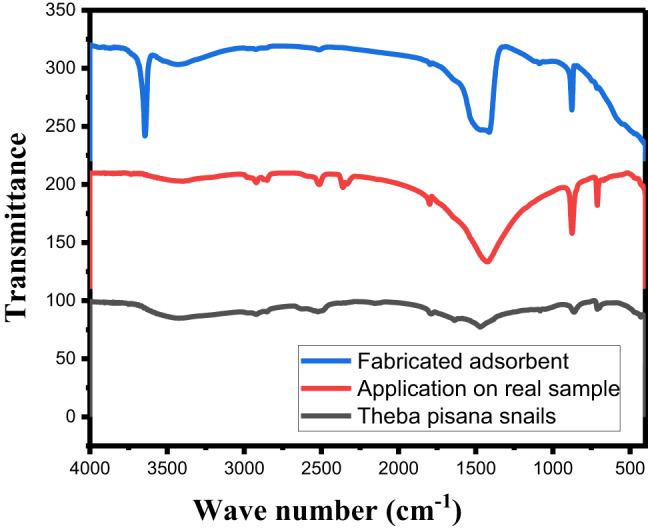
FT-IR spectra of calcined snails before and after lead and ferrous ions adsorption in range of wavenumber from 4000 cm^-1^ to 400 cm^-1^.

Figure 3 displays the FT-IR spectra of Theba pisana snails and the manufactured adsorbent both before and after adsorption. A distinct and broad peak in the wavelength range between 3637.88 cm^−1^ was observed in the CaO derived from snails, as shown in Fig. 3, suggesting vibration of the O–H functional groups present in the CaO structure. Furthermore, specific spikes in the 1468, 1051, and 872 cm^−1^ range were discovered, confirming the C = O and C–O–O functional group vibrations, respectively. CaO NPs have carbonate (CO_3_)^−2^ spectra at 872 cm^−1^ (Fig. 3). The strength of the CaO NPs band, which indicates the stretching of the C–H bond, suggests that the CaO NPs are carbonized at 1468 cm^−1^, 3638 cm^−1^, and 872 cm^−1^.

A peak was observed at 3642 cm^−1^, which is consistent with the hydroxide’s hydroxyl group stretching peak for CaO made from eggshells, suggesting that the O–H functional groups in the CaO structure are vibrating. CaO NP removed lead and iron by a number of methods, including the presence of oxides, hydroxides, and carbonates, among other surface sediments. Ions were able to adsorb by means of surface hydroxyl bonding and isocrystalline replacement with Ca^2+^ ions. FTIR spectra also show that during the adsorption process, Ca^2+^ ions may exchange ions with Pb (II) and Fe (II) and experience an isomorphic mass exchange reaction^54^. Snails and calcined snails’ FT-IR spectra before and after adsorption are displayed in Fig. 3.

#### BET and Particle size distribution

**Fig. 4:**
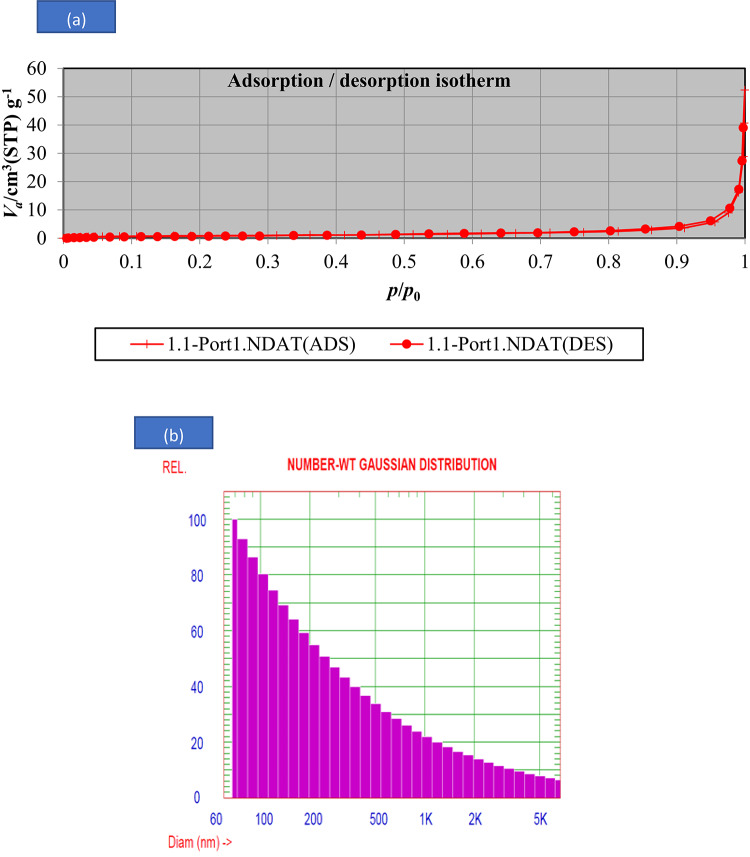
(**a**)N_2_-adsorption–desorption isotherm for prepared nanocomposite, the inset graph refers to the pore size and (**b**) Particle size distribution for prepared adsorbent under study.

The N_2_ adsorption–desorption isotherms and pore size distribution curves of prepared adsorbent were presented in Fig. 4a. According to the International Union of Pure and Applied Chemistry (IUPAC) classification, the isotherms were equivalent to Type-IV isotherms, which was an indicative of the presence of a mesoporous structure and abundant slit pores on the surfaces^55^. A type H_3_ hysteresis loop was clearly observed in the isotherms which revealed that their pore networks were composed of macropores that were not completely filled with pore condensate. As summarized in Table S5, the BET surface area, total pore volume and average pore size for prepared adsorbent were 3.5891 m^2^ g^−1^, 0.02468 m^3^ g^−1^ and 27.506 nm, which proved that adsorbent produced by calcination of Theba pisana had surface area and a good porosity.

The particle size distribution for adsorbent is roughly approximated by Gaussian, as seen in Fig. 4b GAUSSIAN SUMMARY; Mean Diameter = 75.9 nm; Variance (P.I.) = 22.982, Standard Deviation = 363.7 nm (479.4%); Chi Squared = 1.709, Norm. Standard Deviation = 4.794 Baseline Adj. = 0.000%, (Coeff. of Var’n) Z-Avg. Diff. Coeff. = 6.92E-009 cm^2^/s.

## Adsorption studies

### Factors controlling the adsorption process

#### Effect of PH

**Fig. 5:**
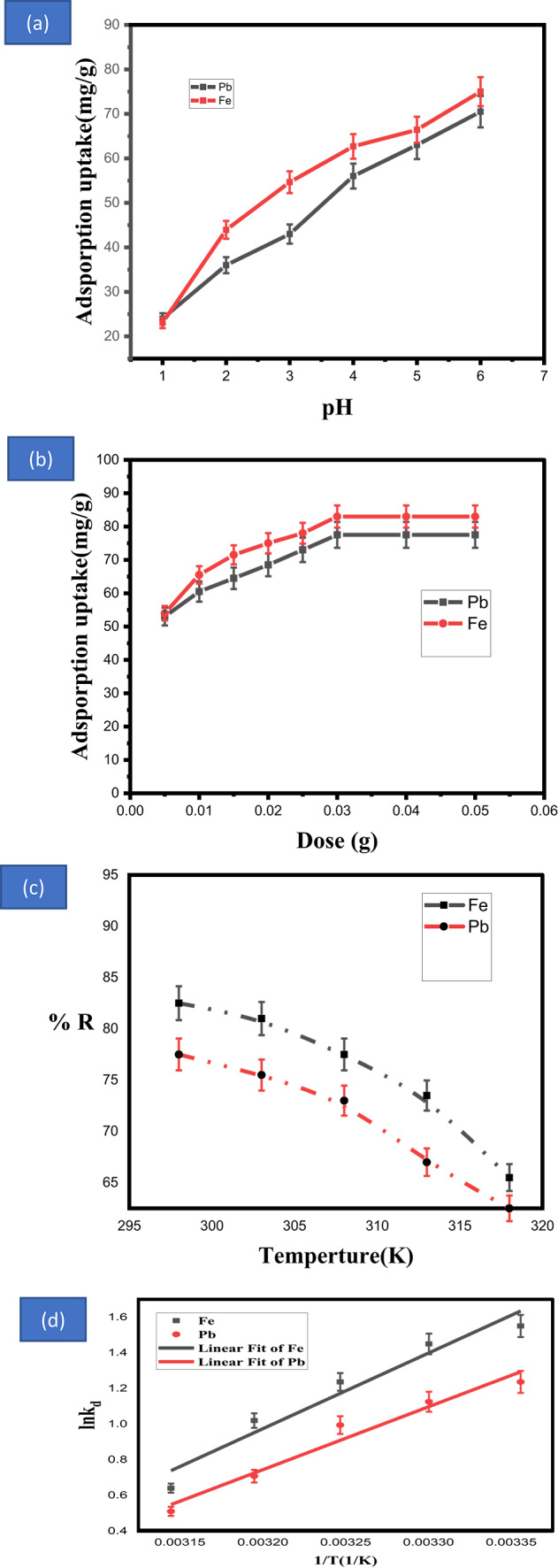
(**a**)Impact of PH on the adsorption efficiency (%) of lead and ferrous ions ,(**b**) Effect of dosage of fabricated adsorbent on adsorption of lead and ferrous ions, (**c**)Effect of temperature and (**d**)Van’t Hoff (Thermodynamics of adsorption process) plot on adsorption process of lead and ferrous ions onto calcined adsorbent (dose = 0.03g, pH = 5, volume of solution = 15 mL, and agitation speed = 200 rpm).

The adsorption of metal ions is greatly affected by pH as impacts the shape; surface charge; and degree of hydrolysis of the metal ions in the solution^49–52^. Because of the different chemical characteristics of the lead and ferrous ions and how they interact with the sorbent surface, there are reported variations in the effectiveness of metal ion removal. Figure 5a shows the impact of pH of solution on the adsorption efficiency of Fe (II) and Pb (II) by the fabricated adsorbent. The removal efficiency increasing as value of pH increasing from 1 to 6. There is a low efficiency at low value of pH. The low adsorption efficiency may due to the competition between the metal ions and the hydronium ions. As the pH rises, the prepared adsorbent’s surface has fewer positive active sites, which lessens their electrostatic repulsion with iron and lead ions^56^. The highest adsorption was seen at pH 6 for Fe (II) and Pb (II); indicating that this was the ideal pH. at order to link metal removal to the adsorption process, all studies were performed at the pH range of 1–6, which prevents chemical precipitation^57^. To prevent precipitation, the following steps were taken at pH = 5.

#### Effect of adsorbent weight

To examine the impact of adsorbent weight on lead and iron metal ions adsorption effectiveness, the adsorbent dose was varied in the sorption cycle from 0,005 to 0.05 g (0.005, 0.01, 0.015, 0.02, 0.025, 0.03, 0.04, 0.05 g), as shown in Fig. 5b. The initial lead and iron concentration in each of these tests was 200 mg/L in a 15 mL solution with a pH of 5 and a temperature of 298 K for 120 min. As the dosage of adsorbent increases, the adsorption efficacy improves rapidly because a higher adsorbent weight results in a larger surface area accessible for adsorption process.

Conversely, when the adsorbent weight increased, the adsorbed quantity reduced because the relative ratio of the adsorbed concentration of lead and ferrous ions to the adsorbent weight (C_0_–C_e_)/m dropped (Eq. 7). However, when adsorbent dose increases, the quantity of metal ion adsorption in each gram of adsorbent, q_e_ (mg /g), drastically drops as shown in Fig. 5b.

A mathematical explanation and illustration of the inverse connection between adsorbent doses (W) and metal ion absorption (q_e_). An inverse connection between metal ions adsorption (q_e_) and adsorbent doses (m) is shown by the provided equation. This effect was mathematically described by using the following equation^58^:

7 $${\varvec{q}}_{{\varvec{e}}} = \frac{{\left( {\% \times {\varvec{C}}_{{\varvec{O}}} } \right)}}{{100 \times {\varvec{m}}}}$$

The absorption capacity (q_e_) value decreases as the removal efficiency (%) rises with the adsorbent since the concentration remains constant.

#### Effect of temperature and thermodynamic nature

Another crucial adsorption parameter is temperature, which establishes whether the process is exothermic or endothermic. The current study examined the influence of temperature on removal efficiency of adsorption between 298 and 318 K (Fig. 5c). It is clear that measurements were made throughout time to determine how temperature affected the adsorption of lead and iron by the calcined adsorbent. The findings demonstrate that adsorption capacity increased as temperature rose, suggesting that the adsorption process for lead and iron was exothermic. This implies that lower temperatures encourage lead and iron molecules to migrate from the adsorbate liquid to the calcined adsorbent solid phase, which lowers the amount of metal in wastewater. Moreover, higher temperatures prevented lead and iron molecules from migrating from the bulk solution to the surface of calcined adsorbent and its pores. The adsorption of lead and iron on the adsorbent surface may be reduced by raising the temperature^59,60^.

Table 1 and Fig. 5d Van’t Hoff display the thermodynamic characteristics of the adsorbed ions utilizing the adsorbent. Strong contact occurred throughout the adsorption process of lead and ferrous ions due to large negative values of ΔG^◦^, and the negative values of ΔG^◦^ validated the spontaneity of the metal ion adsorption process. The ΔG^◦^ verified that the adsorption process of lead and ferrous ions was more spontaneous at lower temperatures. As a result, adsorption of metal ions was preferred at room temperatures. It is implied that physical adsorption occurs and is exothermic by the low negative value of ΔH^◦^. For physical adsorption; the ΔH^◦^ value is often less than 80 kJ mol^−1^^61^. In addition to supporting the linear Langmuir isotherm model being the best match adsorption of lead and ferrous ions, the positive values of ΔH^◦^ show that the adsorption process is monolayer coverage. When lead and ferrous ions are adsorbed using calcined adsorbent, the randomness at the solid-solution interface diminishes, as shown by the negative value of ΔS^◦^ (ΔS^◦^ < 0)^62,63^^.^ The adsorption process was exothermic, favoring low temperatures as the temperature increased, according to the negative activation energy (E_a_)^64^. Table 1 displayed the thermodynamic characteristics of metal ion adsorption using an adsorbent.

**Table 4:** Comparison with other adsorbents.

Adsorbent	Analyte	Capacity, mgg^−1^	Ref
Coconut Shells	Pb (II)	11.9	^76^
TC-AC	Pb (II)	47.17	^77^
TT-AC	Pb (II)	46.95	^77^
Ailanthus excelsa bark biosorbent	Pb (II)	22.72	^78^
Biochar from elephant grass	Pb (II)	13.80	^79^
CaO NPS	Pb(II)	77.5	This study
Sepiolite	Fe (II)	12	^80^
Ca-Pal	Fe (II)	3.71	^81^
PSBAC	Fe (II)	41.66	^82^
Polyaniline-coated sawdust	Fe (II)	31.56	^83^
Chitosan-tripolyphosphate bead	Fe (II)	11.65	^57^
CaO NPS	Fe (II)	82.5	This study

#### Time effect and kinetic of adsorption process

**Fig. 6:**
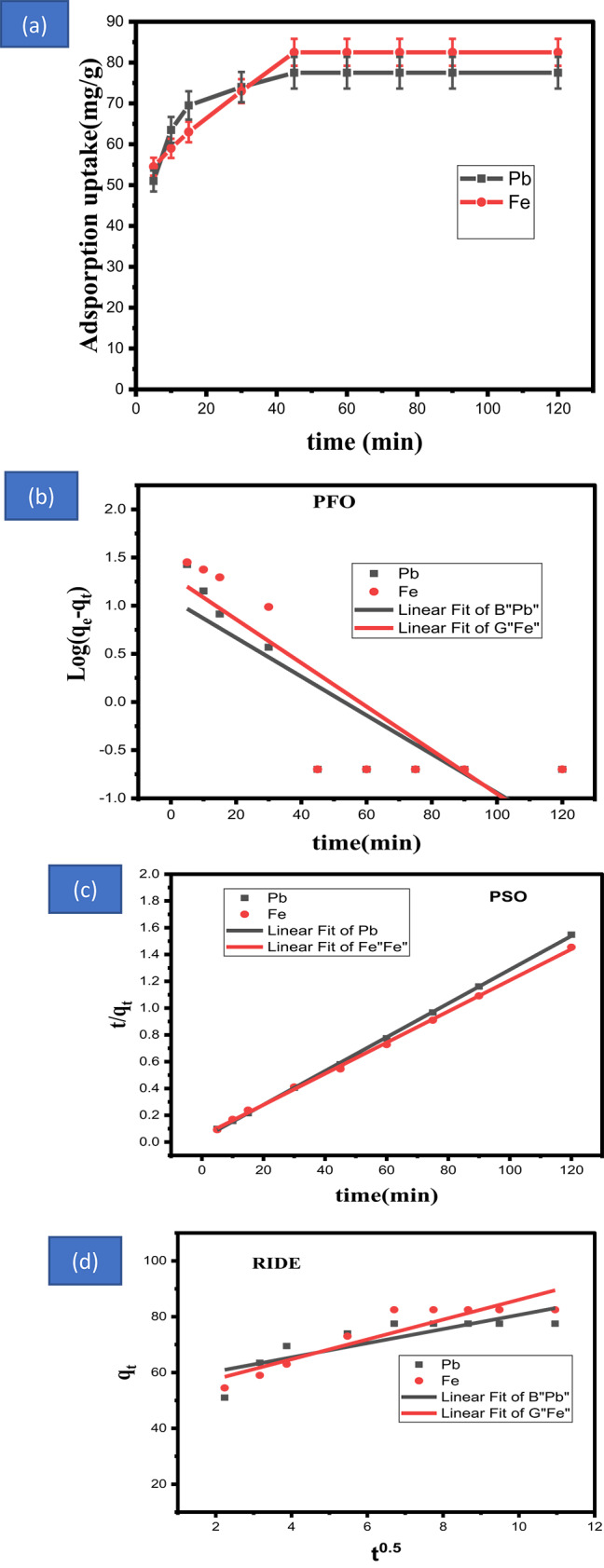
**(a)** Effect of time on adsorption process, (**b**), (**c**) and (**d**) Adsorption kinetic parameters of adsorbent on the recovery of metal ions.

One important component of HMI adsorption onto an adsorbent is the time effect. The findings of an investigation into the impact of time are shown in Fig. 6a. Adsorption capacity and efficiency rise steadily over time, reaching their peak levels at 45 min. Adsorption at the first stage of ion adsorption is made possible by the adsorbent sites’ larger surface area and free binding sites. This capability grows over time until the sites are saturated at 45 min and stays constant after that. The sorption rate is controlled by the speed at which HMI adsorbate migrates from the outside to the inside of the adsorbate molecules. Ultimately, an equilibrium time of 45 min was selected and used for the remaining studies.

### Adsorption kinetics

The kinetics of lead and iron adsorption by absorbent must be understood in order to calculate the rate of MI adsorptive removal from aqueous medium^49–52^ Additionally, this information offers important insights regarding the adsorption process. To describe the process of adsorption, the following models were taken into consideration: pseudo first order, pseudo second order, and intraparticle diffusion^65^. Table 2 and Fig. 6b–d contains the linear formulae and associated parameters for various kinetic models. The elements of the PFO and PSO models were carefully assessed, and Table 2 provides a summary. The PSO model is well-aligned with the adsorption of HMI by the adsorbent, as demonstrated by Table 2, due to the good agreement between the predicted and observed qe values. Additionally, the PSO model’s R^2^ values are somewhat greater than the PFO models. Additionally, Table 2’s absolute percentage error (RSS and Pearson’s) indicates that the best model fits are provided by PSO, which has the lowest RSS value. According to these findings, lead and iron is chemisorbed onto an adsorbent^66^.

### Effect of concentration of lead and ferrous ions and isotherms

**Fig. 7:**
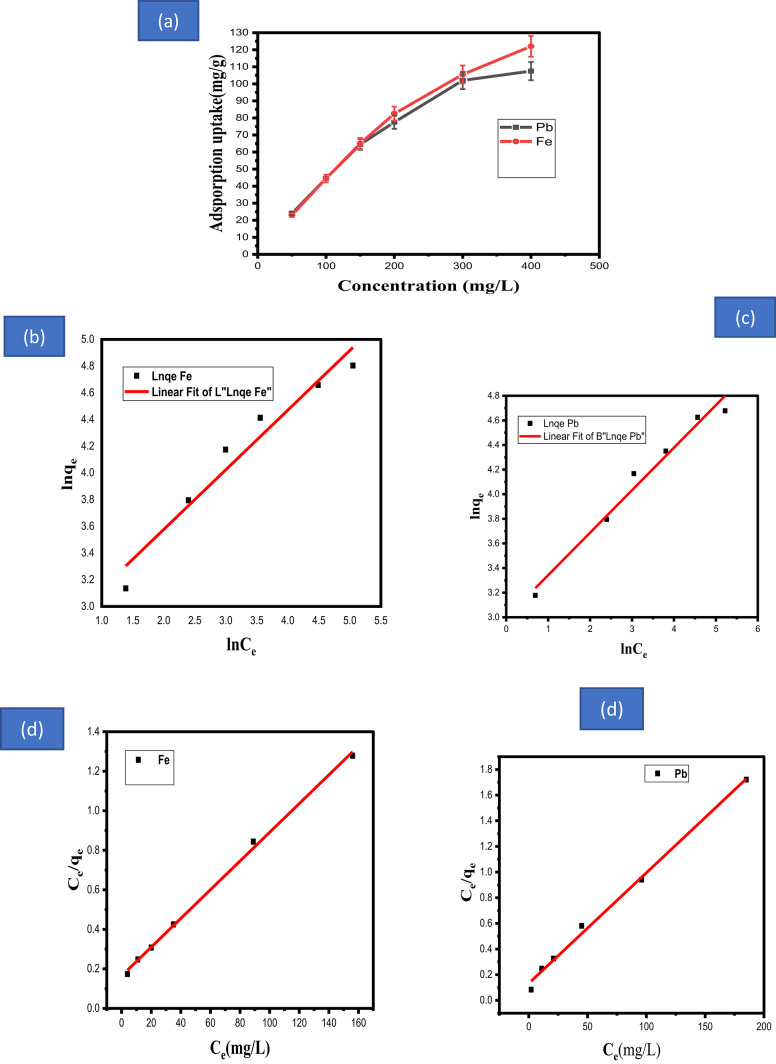
(**a**) Effect of concentration, (**b**), (**c**)Freundlich isotherm for adsorption of lead ferrous ions on prepared calcined adsorbent, (**d** and **e**) Linear Langmuir isotherm for lead and ferrous ions adsorption on prepared calcined adsorbent.

A major influence on the adsorption process is the initial concentration of metal ions. Figure 7a displayed the impact of changing the HMI concentrations (50–400 mg/L) on the adsorption uptake and efficiency. The experiment was performed for 45 min at temperature of 25 °C with a fixed dosage of calcined adsorbent (0.03 gm) in 15 mL of a solution with a starting pH of 5 for lead and iron. As the ion concentration rises, the adsorption effectiveness falls. The mass transfer resistance of lead and ferrous ions is not overcome by raising the concentration of ions, when the amount of lead and ferrous ions increases with the same mass of calcined adsorbent^67,68^. However, when the concentrations of both metal ions rise from 50 mg/L to 400 mg/L, lead and iron adsorption capability on the calcined adsorbent rises.

Understanding the adsorption potential of the calcined adsorbent and its affinity for the HMI adsorbate requires knowledge of the adsorption isotherm^69^.

To identify the most suitable adsorption model among the five tested (Freundlich, Langmuir, and others), the experimental data were fitted to different adsorption isotherms. The graphical findings are displayed in Fig. 7b–d, and the isotherm models’ fitting parameters are listed in Table 3. The findings indicate that the adsorption isotherm of lead and iron adsorbate by adsorbent follows Langmuir rather than Freundlich, based on the correlation coefficients. The values of n for lead and ferrous ions from the Freundlich isotherm are used to quantify the favorable adsorption process and are correlated with it. Given that the n values for lead and ferrous ions are given in Table 3, the adsorption is advantageous when n falls between 0 and 10. Since the Freundlich exponent value is greater than 1, it indicates physical adsorption for both lead and ferrous ions^70^. may be used to determine the separation factor (R_L)_, which establishes the affinity between the calcined adsorbent and adsorbate.

8 $${\varvec{R}}_{{\varvec{L}}} = \frac{1}{{\left( {1 + {\varvec{K}}_{{\varvec{l}}} {\varvec{C}}_{{\varvec{O}}} } \right)}}$$

The Langmuir model’s K_l_ value was used to determine the R_L_ value, which is crucial for determining the isotherm’s favorability. The isotherm’s shape is favorable if the R_L_ value falls between 0 and 1, linear if R_L_ = 1; unfavorable if R_L_ exceeds 1; and irreversible if R_L_ = 0^49–51^. According to Table S6, the calculated separation factor for MI (Fe, Pb) onto the adsorbent was less than 1 but larger than zero, indicating good adsorption. Additionally, the adsorption process is beneficial because R_L_ constant in the linear Langmuir and (1/n) of linear Freundlich isotherms for lead and ferrous ions is achieved to be less than 1^71^.

## Desorption studies

**Fig. 8:**
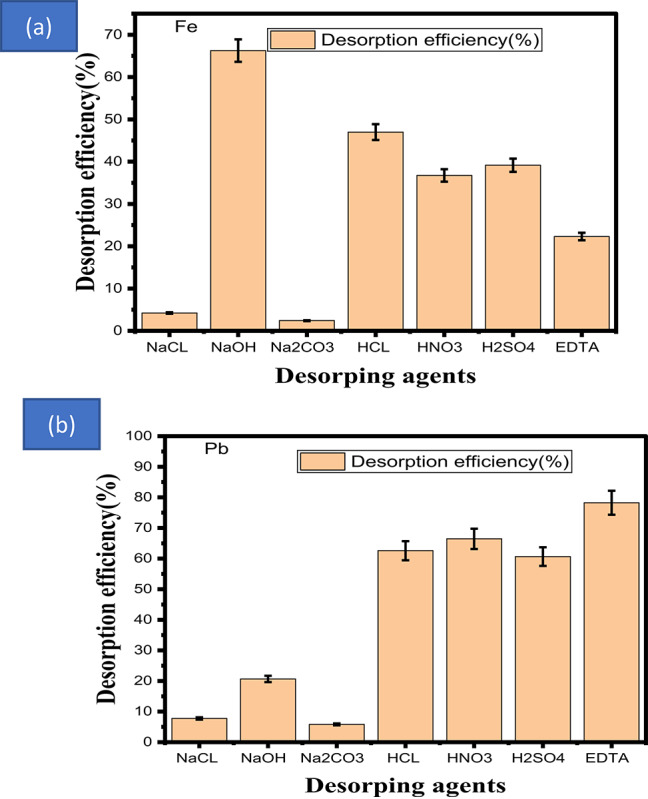
(**a**) and (**b**) Eluting agents for different metal ions.

The sorbent type and the underlying sorption mechanism both have a substantial impact on the desorption process’s efficiency, which in turn affects the selection of a suitable washing solution. The sorbent material must not be harmed by the washing solution, which must also be inexpensive, efficient, and ecologically beneficial. The sorbent type and the underlying sorption mechanism both have a substantial impact on the desorption process’s efficiency, which in turn affects the selection of a suitable washing solution. The sorbent material must not be harmed by the washing solution, which must also be inexpensive, efficient, and ecologically beneficial^72^ Because it makes water treatment more cost-effective, the ability to reuse adsorbent materials is a crucial trait that has to be studied^72^. The washing solution needs to be efficient, cost-effective, safe for the environment, and not harmful to the sorbent material. Figure 8a–b displays the most effective eluting agents for various metal ions. We conducted follow-up studies to investigate the feasibility of employing the calcined adsorbent in consecutive adsorption–desorption cycles after determining the amount of time needed for the desorption process to reach saturation. Due to the elimination of the metal ions evocative of the first cycle, the desorption efficiency, which was high in the first cycle, improves in the second and third cycles before gradually declining. The produced material is an adsorbent, according to these results. Furthermore, there was no discernible particle deformation, dissolution, or fragmentation as a result of the desorption process. The particles’ stability and physical integrity were preserved over the course of the repeated cycles, indicating that they are suitable for reuse under the given desorption circumstances.

### Expected mechanism

Different chemical interactions and surface characteristics may be involved in the reaction mechanism, which enhances the effectiveness of the adsorption process.

1. Physical adsorption: Through electrostatic interactions and weak van der Waals forces, metal ions attach to the CaO NP surface via physical adsorption. CaO NPs’ tiny particle size and large surface area facilitate this process by increasing the number of adsorption-active sites. For example, non-specific interactions allow lead and ferrous ions to stick to the surface of CaO. Because van der Waals forces are modest, the process is usually reversible, and the adsorption capacity is temperature-sensitive.
2. Ion exchange: In this process, Ca^2+^ ions on the surface of CaO nanoparticles are swapped out for metal ions (lead and ferrous ions) in the aqueous solution. Because of their comparable ionic radii and charge densities, heavy metal ions like Pb (II) and Fe (II) can interchange with Ca (II) High selectivity for metal ions, for example, is a characteristic of the ion exchange process; selectivity coefficient values show how well various metal ions are exchanged. With exchange capacities ranging from 1.5 to 3.0 mmol/g based on the metal ion and CaO surface characteristics, this process can be very useful in eliminating hazardous heavy metals from aqueous solutions.
3. Complexation of the surface: When metal ions come into contact with reactive locations on the surface of CaO NPs, like oxygen atoms (–O–) or hydroxyl groups (–OH), surface complexation takes place. There are two types of complexation in this mechanism: inner-sphere and outer-sphere^73–75^.

In inner-sphere complexation, metal ions directly coordinate with surface groups to generate strong chemical interactions. For example, when lead ions (Pb (II)) form inner-sphere complexes with hydroxyl groups on the surface of CaO, lead (II) hydroxide (Pb(OH)₂) can develop and precipitate out of the solution. Strong binding is indicated by this reaction’s high stability constant (log K ∼ 6) for the creation of these complexes.

On the other hand, because outer-sphere complexation involves weaker electrostatic connections where water molecules remain between the lead and ferrous ions and the surface of calcined adsorbent, it leads to less persistent adsorption. The small particle size and high density of reactive sites on CaO NPs enhance these interactions.

**Fig. 9:**
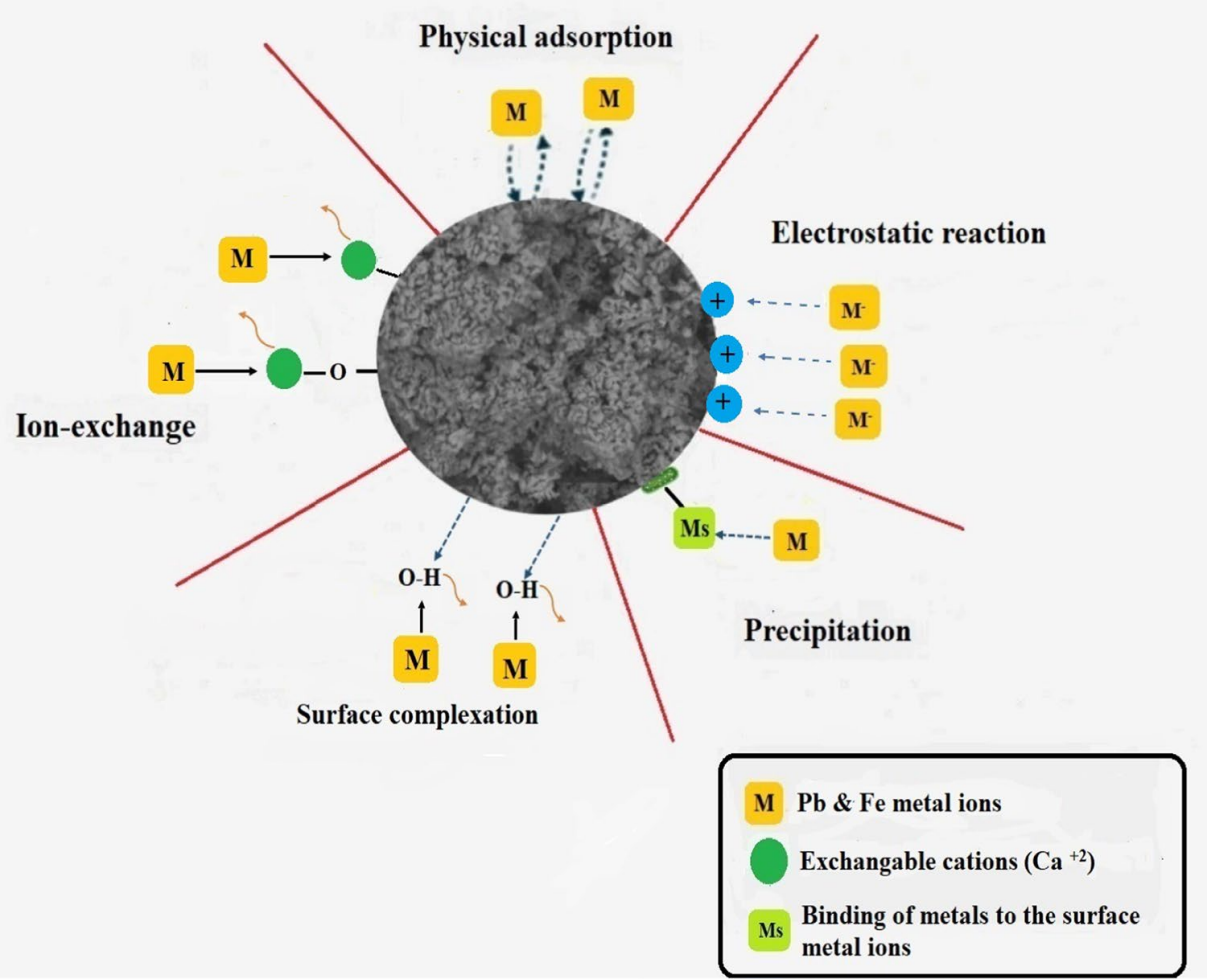
Expected Mechanism for adsorption of lead and ferrous ions with prepared calcined adsorbent.

(4) (4) Precipitation: When CaO NPs interact with the lead and ferrous ions in the solution, insoluble metal hydroxides or carbonates are produced. For example, Pb(OH)₂ and chromium (III) Fe(OH)₂ are metal hydroxides that precipitate out of the water when Pb (II) react with hydroxide ions (OH⁻) in the solution. Furthermore, if carbonate ions (CO₃^2⁻^) are present; lead and ferrous ions can react with them to create metal carbonates, such as PbCO₃, FeCO₃, and ₃, which precipitate and extract the lead and ferrous ions from the solution. These chemicals’ poor solubility products (Ksp) are what cause these precipitates to develop. For example, Pb (OH)₂ has a very low solubility product ($KSP \sim 1.2\times {10}^{-15}$), which causes a lot of precipitation and removes Pb (II) ions from the solution. The expected Mechanism for adsorption of lead and ferrous ions with prepared calcined adsorbent is shown in Fig. 9.

## Application (Real wastewater)

Adsorption tests were conducted using real waste samples gathered from labs to further examine the efficacy of the calcined adsorbent in real-world applications. The chemical composition of the waste sample was shown in Table S7. As a case study, the produced adsorbent was used on an actual sample. The fluid that is produced is exposed to the ideal adsorption conditions, which include a 45-min adsorption duration and a pH of 6 ± 0.1 to prevent precipitation. ICP-MS and UV–VIS spectrophotometer are used to quantify the concentration of ions in solutions. The efficacy of the calcined adsorbent for lead and ferrous ions adsorption from both synthetic and actual contaminated liquor solutions was demonstrated by the genuine sample’s high adsorption efficiency. Adsorption of the same metal ions is easier at low concentrations than at greater concentrations. The outcomes of employing the calcined adsorbent for MI adsorption show that it functions effectively as an adsorbent for lead and ferrous ions sorption ) SEM micrograph images and EDX spectra of loaded fabricated adsorbent after adsorption and application to real waste solutions are shown from Fig. 2e–h.

### Comparisons for the adsorption process

The optimization of lead and iron adsorption onto prepared adsorbent was performed considering the factor s of fabricated adsorbent pH (1–6); concentrations (50–400 mg/L); adsorbent dose (0.005–0.05g), time (5–120 min) and temperature (298–318K). The optimum conditions for the prepared adsorbent was found at 45 min, pH = 6 and at room temperature. Comparisons for the adsorption process of lead and iron using different adsorbents is summarized in Table 4 Furthermore, as indicated in Table 4, the adsorption capabilities of the metal ions acquired in this investigation have been contrasted with those of earlier investigations. Surprisingly, Table 4 demonstrated that CaO NPs had a greater adsorption capacity than the other adsorbents from the literature.

## Conclusion

In our study, we describe the synthesis and properties of an adsorbent derived from the terrestrial snail T. pisana that was employed to extract of lead and ferrous ions from aqueous solutions. SEM–EDX, DLS, BET, and FTIR results were used to characterize the prepared calcined adsorbent in order to evaluate its the morphological and functional groups. The SEM images demonstrated how permeable the pores were on fabricated adsorbent’s surfaces and BET demonstrated also mesoporous of it. The morphologies of the adsorbent were changed before and after Adsorption was verified by modifications in experiments. Batch studies were carried out to know the different factors affect the adsorption process. The batch approach determined the optimal sorption conditions, including a pH 6, and 45 min of sorption time. The sorption process for adsorbent was described by many linear isotherms and kinetic models. . PSO and Langmuir successfully describe the adsorption kinetics and isotherms. The adsorption process is spontaneous, exothermic, and random-increasing, according to the values of ΔG^o^, ΔH^o^, and ΔS^o^. We demonstrated through thermodynamic studies that the adsorbent’s removal of MI molecules is an exothermic, spontaneous process. The adsorbent may be effectively reused in a minimum of six successive adsorption–desorption cycles, as we have finally demonstrated. It is confirmed that the developed adsorbent is a promising material for the removal of lead (II) and ferrous (II) ions dissolved in aqueous medium when the adsorbing performance of this composite is compared to other materials. These findings underscore the promising potential of using prepared adsorbent from snails for efficient lead (II) and ferrous (II) ion extraction from aqueous solutions and for treating and recovering low concentrations of metal ions containing waste water. An apparent plan for the development of metal ion removal technology using land snail pests and an environmental approach is presented in this paper. It outperformed the prepared samples that were available in terms of metal ion uptake.

## Supplementary Information

Below is the link to the electronic supplementary material.

Supplementary Material 1

## Data Availability

No datasets were generated or analysed during the current study.
