# Supplementary material for: Synthesis of ecologically effective adsorbent from theba pisana snails for enhanced adsorption of Pb and Fe
Source: Sci Rep. 2025 Nov 11;15:39416. doi: 10.1038/s41598-025-22770-0 (PMC12606130; doi:10.1038/s41598-025-22770-0)
Supplement: Supplementary file 1 — Supplementary Material 1 [file 41598_2025_22770_MOESM1_ESM.docx]

**Supplementary file**

**Synthesis of Ecologically Effective Adsorbent from Theba pisana Snails for Enhanced adsorption of Pb and Fe**

Amany R. Salem^1^*, Ibrahim Hegazy^2^, Walaa A. Kassab ^1^, Hesham A.M. Ibrahim^3^

^1^Nuclear Materials Authority, El -Maadi, P.O. Box 530, Cairo, Egypt

^2^ The Holding Company for Drinking Water in Greater Cairo (10th of Ramadan Authority), Cairo, Egypt

^3^Department of Agricultural Zoology and Nematology, Faculty of Agriculture, Al-Azhar University, Assiut Branch, Assiut, 71524, Egypt

***Corresponding author email:** [chem_amany@yahoo.com](mailto:chem_amany@yahoo.com) ;[dr_amanyragab_nma@yahoo.com](mailto:dr_amanyragab_nma@yahoo.com)

https://orcid.org/0000-0002-2269-6702

(a)


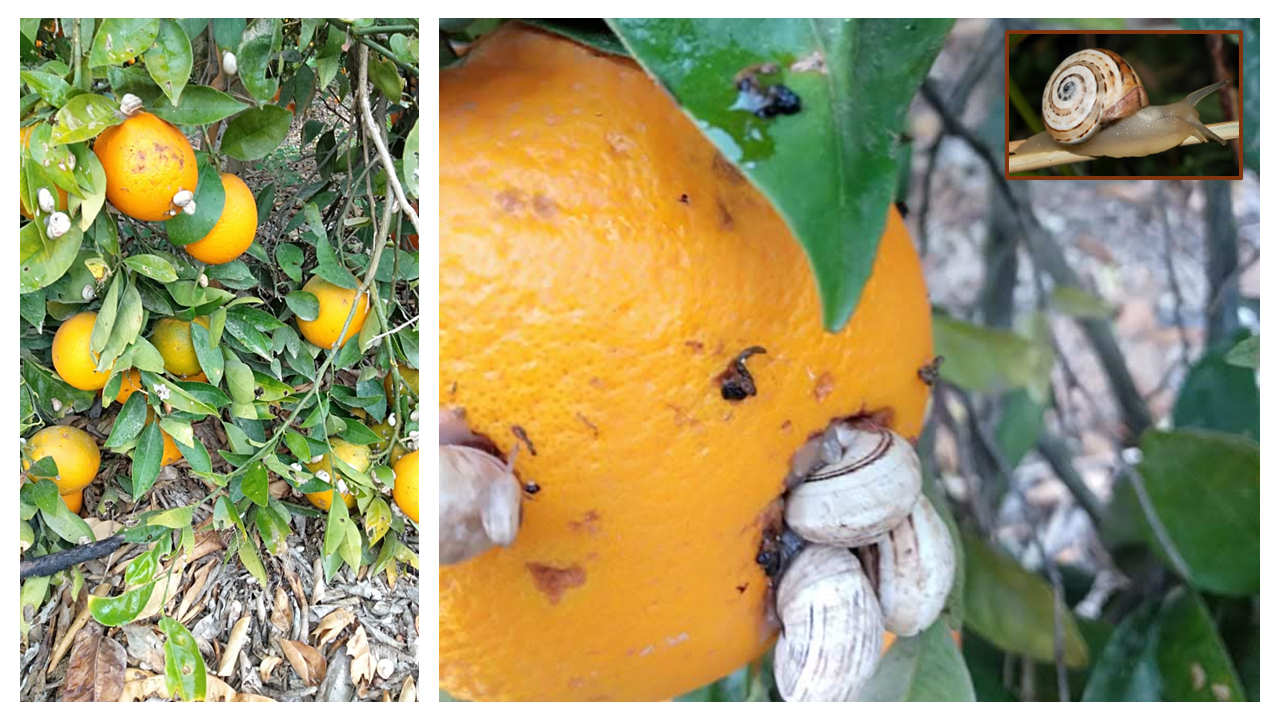


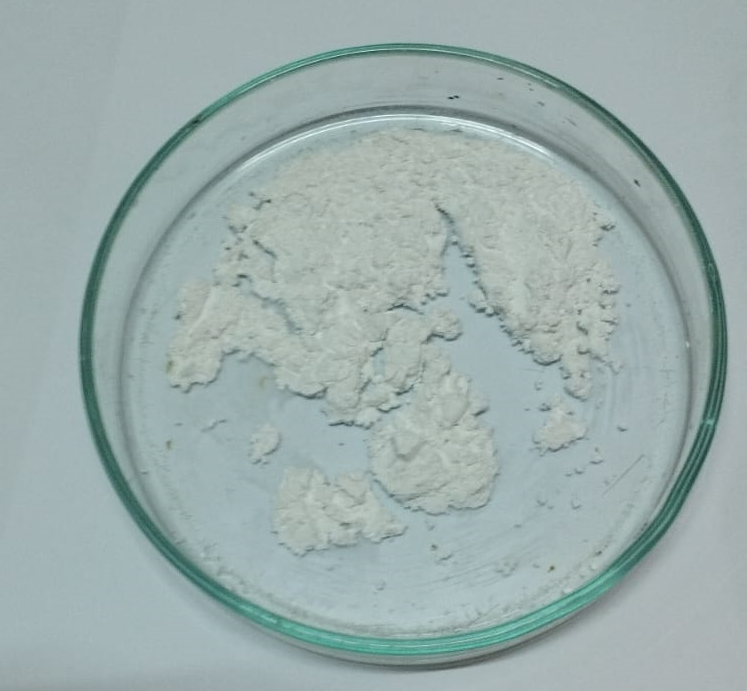


(b)

**figure S1:** (a) individuals of the land snail Theba pisana on infested trees and the damage they cause and (b) a photograph of the white garden snail calcium oxide

**Table S1:** EDX analysis of Theba pisana Snail

| Element | Atomic % | Atomic % Error | Weight % | Weight % Error |
| --- | --- | --- | --- | --- |
| C | 27.0 | 0.3 | 17.8 | 0.2 |
| N | 14.9 | 2.1 | 11.5 | 1.6 |
| O | 41.5 | 1.1 | 36.4 | 0.9 |
| Na | 1.4 | 0.1 | 1.7 | 0.1 |
| Al | 0.2 | 0.0 | 0.3 | 0.0 |
| Si | 0.3 | 0.0 | 0.4 | 0.0 |
| P | 0.3 | 0.0 | 0.6 | 0.0 |
| S | 0.4 | 0.0 | 0.8 | 0.0 |
| Cl | 0.9 | 0.0 | 1.8 | 0.0 |
| K | 0.4 | 0.0 | 0.8 | 0.1 |
| Ca | 12.8 | 0.1 | 28.1 | 0.3 |

**Table S2**: EDX analysis of Theba pisana after thermal effect

| Element | Atomic % | Atomic % Error | Weight % | Weight % Error |
| --- | --- | --- | --- | --- |
| C | 5.1 | 0.1 | 2.6 | 0.1 |
| O | 62.6 | 0.9 | 42.4 | 0.6 |
| Ca | 32.4 | 0.2 | 55.0 | 0.3 |

**Table S3:** EDX analysis of the loaded fabricated adsorbent after adsorption and application to

a real waste sample

| Element | Atomic % | Atomic % Error | Weight % | Weight % Error |
| --- | --- | --- | --- | --- |
| C | 9.3 | 0.2 | 5.5 | 0.1 |
| N | 10.6 | 1.8 | 7.3 | 1.2 |
| O | 59.4 | 0.9 | 46.9 | 0.7 |
| Na | 0.4 | 0.1 | 0.4 | 0.1 |
| Mg | 0.0 | 0.0 | 0.0 | 0.0 |
| Al | 0.2 | 0.0 | 0.2 | 0.0 |
| Si | 0.2 | 0.0 | 0.2 | 0.0 |
| P | 0.2 | 0.0 | 0.3 | 0.0 |
| S | 0.4 | 0.0 | 0.6 | 0.1 |
| Cl | 0.2 | 0.0 | 0.3 | 0.0 |
| K | 0.0 | --- | 0.0 | --- |
| Ca | 19.1 | 0.2 | 37.8 | 0.3 |
| Ti | 0.0 | --- | 0.0 | --- |
| Fe | 0.1 | 0.1 | 0.4 | 0.2 |

**Table S4**: Another EDX analysis of the loaded fabricated adsorbent after adsorption and application to a real waste sample

| Element | Atomic % | Atomic % Error | Weight % | Weight % Error |
| --- | --- | --- | --- | --- |
| C | 8.1 | 0.2 | 4.0 | 0.1 |
| N | 12.4 | 1.3 | 7.1 | 0.8 |
| O | 57.7 | 0.8 | 37.8 | 0.6 |
| Na | 0.6 | 0.1 | 0.6 | 0.1 |
| Al | 0.4 | 0.0 | 0.4 | 0.1 |
| Ca | 17.0 | 0.2 | 28.0 | 0.3 |
| V | 0.5 | 0.1 | 1.0 | 0.1 |
| Mn | 0.4 | 0.0 | 0.8 | 0.1 |
| Fe | 0.6 | 0.1 | 1.3 | 0.2 |
| Pb | 2.2 | 0.1 | 18.9 | 0.6 |

**Table S5:**Surface areas, total pore volumes and average pore sizes of the fabricated adsorbent

| **BET plot** | **units** | **Value** |
| --- | --- | --- |
| Vm | [Cm^3^(STP)g^-1^] | 0.8246 |
| a_s_,_BET_ | [m^2^g^-1^] | 3.5891 |
| C |  | 33.679 |
| Total pore volume(p/p_0_) | [Cm^3^g^-1^] | 0.02468 |
| Average pore diameter | nm | 27.0506 |

**Table S6:** R_L_ at different concentrations of Pb (II) and Fe (II) ions

| **C_o_ (mg/L)** | **Pb (II)** | **Fe (II)** |
| --- | --- | --- |
| 50 | 2.35×10^-1^ | 3.11×10^-1^ |
| 100 | 1.33×10^-1^ | 1.84×10^-1^ |
| 150 | 9.30×10^-2^ | 1.31×10^-1^ |
| 200 | 7.14×10^-2^ | 1.02×10^-1^ |
| 300 | 4.87×10^-2^ | 7.01×10^-2^ |
| 400 | 3.70×10^-2^ | 5.35×10^-2^ |

**Table S7:** Chemical composition of waste sample

| **Analysis type** | **Conc.(mg/L)** | **Analysis type** | **Conc.(mg/L)** |
| --- | --- | --- | --- |
| PH | 6.9 | Lead | 67 |
| C | 27 | Ferrous | 90 |
| sulfide | 8.5 | Zinc | 45 |
| Nitrate | 1.8 | Selenium | 61 |
| Phosphorus | 23 | Phenols | 3.9 |
| Aluminum | 8 |  |  |
